# Uremic Bilateral Breast Calciphylaxis: A Case Report and Literature Review

**DOI:** 10.7759/cureus.46024

**Published:** 2023-09-26

**Authors:** Natalie Hassan, Abdalla Saad Abdalla Al-Zawi, Philip Idaewor, Noreen Rasheed, Dennis Wayne Chicken

**Affiliations:** 1 Critical Care, Mid and South Essex NHS Foundation Trust, Basildon, GBR; 2 General and Breast Surgery, Mid and South Essex NHS Foundation Trust, Chelmsford, GBR; 3 General and Breast Surgery, Anglia Ruskin University, Chelmsford, GBR; 4 Breast Surgery, Broomfield University Hospital, Chelmsford, GBR; 5 Histopathology/Cellular Pathology, Mid and South Essex NHS Foundation Trust, Basildon, GBR; 6 Radiology, Basildon and Thurrock University Hospital, Basildon, GBR; 7 General Surgery, Mid and South Essex NHS Foundation Trust, Basildon, GBR

**Keywords:** mastectomy, skin necrosis, chronic kidney disease, mammogram, calcific uremic arteriolopathy, calciphylaxis

## Abstract

Calciphylaxis, also called calcific uremic arteriolopathy, is a rare benign cutaneous manifestation. Although little is known about its pathogenesis, it is thought to be a result of vascular wall calcification leading to soft tissue necrosis, and it is usually encountered in patients with end-stage kidney disease (ESKD) on long-term renal dialysis. Breast calciphylaxis is a rare entity that may present as a breast mass or necrotic ulcers, and it is common for it to be initially mistaken for a malignant breast pathology. In this article, we present a case of bilateral breast calciphylaxis in a 66-year-old female with ESKD receiving long-term dialysis.

## Introduction

In 1961, Hungarian-Canadian endocrinologist Hans Selye used the term “calciphylaxis” for the first time. He conducted scientific experiments to induce diffuse subcutaneous soft tissue calcification. Certain preparations, such as parathyroid extracts and vitamin D, as well as trauma, were used as triggering factors [1]. His results showed that calcium accumulated in the walls of the arterioles and capillaries of the dermis and subcutaneous adipose tissue, resulting in ischemic necrosis. Calciphylaxis is seen most often in patients with end-stage renal failure requiring dialysis; however, it is also encountered in non-uremic patients [2,3].

## Case presentation

**Figure 1:**
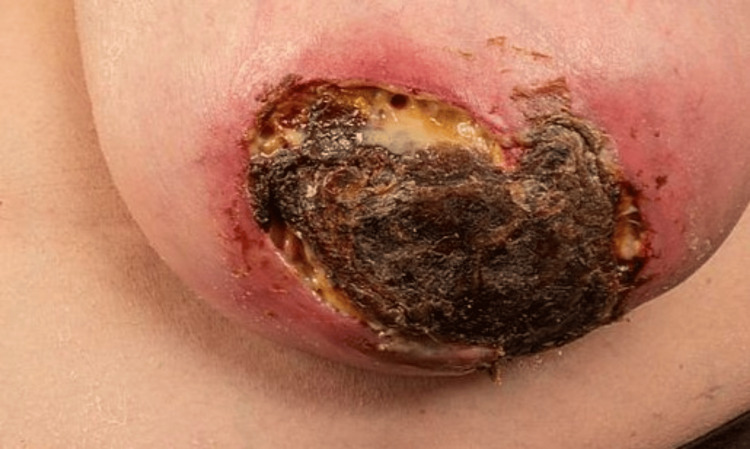
Left breast with a 14 cm ill-defined mass and an 8 cm area of well-demarcated full-thickness skin necrosis.

A 66-year-old female presented with a seven-month history of bilateral painful breast lumps. The triple assessment, including biopsies, showed benign chronic inflammation only. However, she had recently noticed erythematous painful skin changes, which progressed to dark bluish and black skin lesions, followed by discharging skin ulceration in the same areas. She had a background of end-stage kidney disease on regular hemodialysis, and the renal biopsy showed membranous nephropathy. She was also known to have atrial fibrillation, hypertension, depression, migraine, hypercholesterolemia, spondylitis, aortic stenosis, and obstructive sleep apnea (OSA), and she was an ex-smoker. She was on OxyContin, alfacalcidol, epoetin alfa (an erythropoiesis-stimulating agent), tinzaparin (a low molecular weight heparin), Venofer (iron sucrose), and, in the past, Calcichew and calcium acetate. On examination, she had a BMI of 38 and ptotic breasts. In the left breast, there was a large necrotic eschar with underlining sloughy necrotic breast tissue, and deep crevices had developed around the necrotic area (Figure 1).

**Figure 2:**
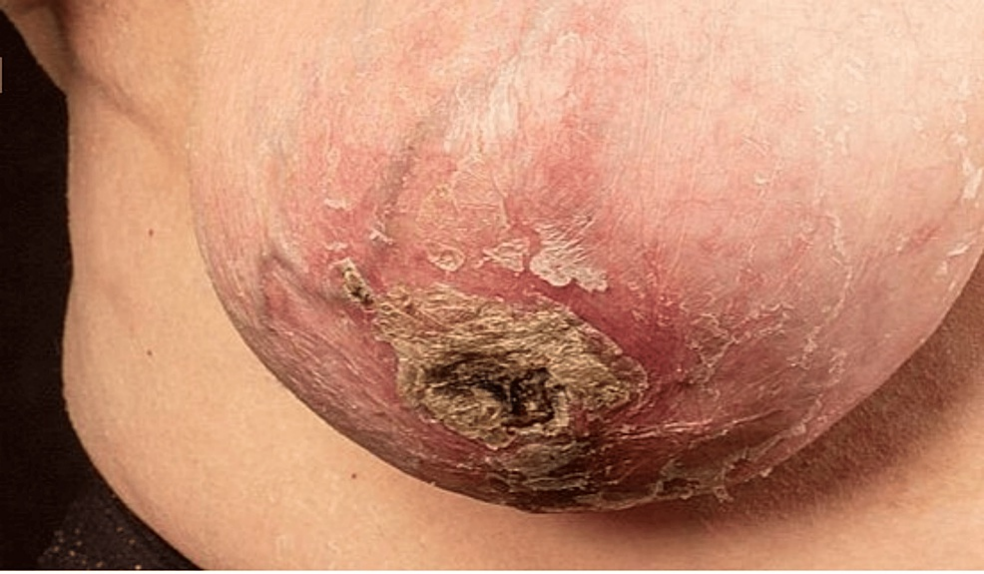
There is a 5 cm area of necrosis on the right breast skin.

On the center of the contralateral breast, there was early skin necrosis (Figure 2).

**Figure 3:**
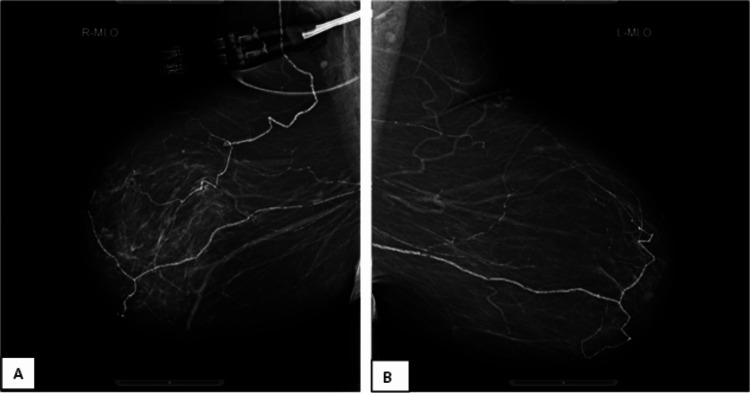
(A) Right mammogram and (B) left mammogram MLO view showing fat-replaced fibroglandular breast parenchyma. No suspicious soft tissue lesions, microcalcifications, or architectural distortions. No mammographic features of malignancy or any significant interval change when compared with the previous screening images done three years earlier. There are significant vascular calcifications bilaterally. MLO: mediolateral oblique.

**Figure 4:**
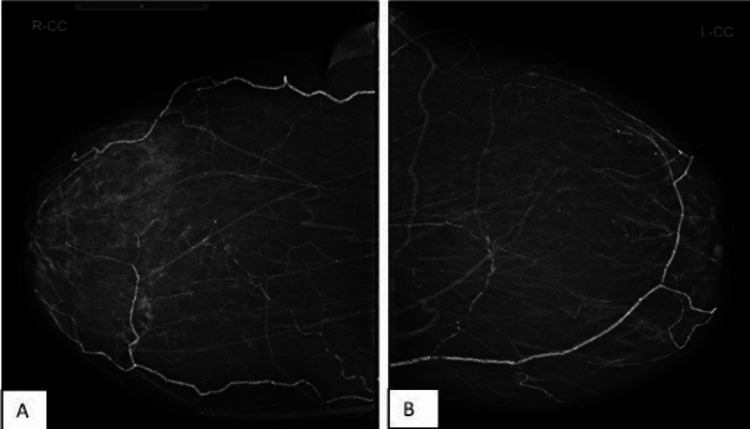
(A) Right mammogram and (B) left mammogram CC view showing fat-replaced fibroglandular breast parenchyma. No suspicious soft tissue lesions, microcalcifications, or architectural distortions. No mammographic features of malignancy or any significant interval change when compared with the previous screening images done three years earlier. There are significant vascular calcifications bilaterally. CC: craniocaudal.

Chronic dry ulceration on the right heel and right first toe was also detected. A blood analysis showed an adjusted calcium level of 2.71 mmol/L (2.2-2.6) and phosphate of 1.72 mmol/L (0.8-1.5). Her mammogram indicated fat-replaced fibroglandular breast parenchyma. No suspicious soft tissue lesions, microcalcifications, or architectural distortions were revealed. There were no mammographic features of malignancy or any significant interval change when compared with the previous screening images done three years earlier. There were widespread significant vascular calcifications bilaterally (Figures 3, 4).

**Figure 5:**
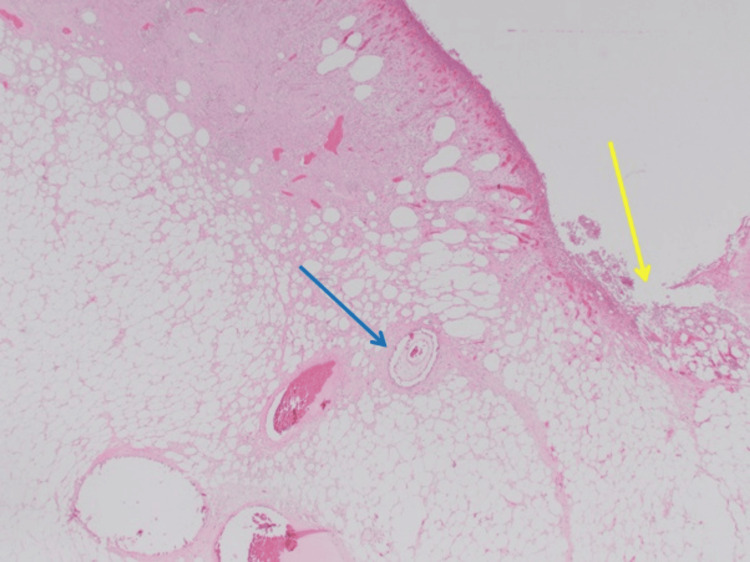
Ulcerated breast skin (yellow arrow) with areas of blood vessel calcification (blue arrow) at x1.25 magnification.

**Figure 6:**
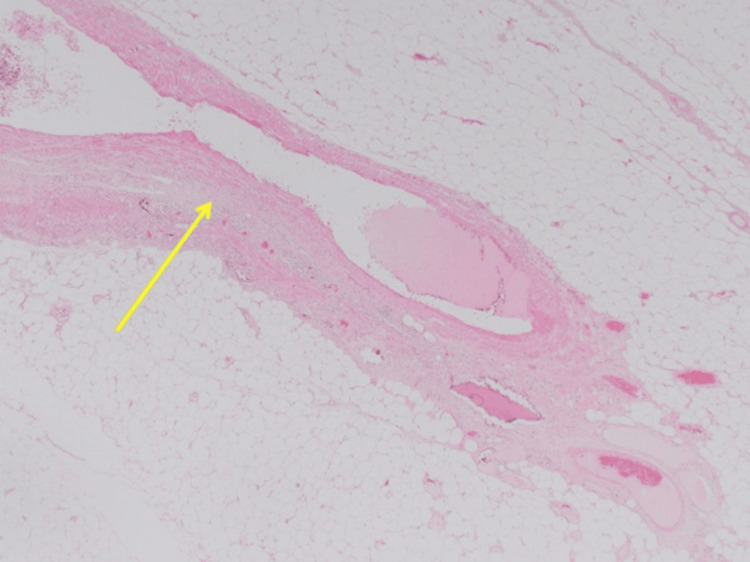
Blood vessel calcification (yellow arrow) at x2 magnification.

**Figure 7:**
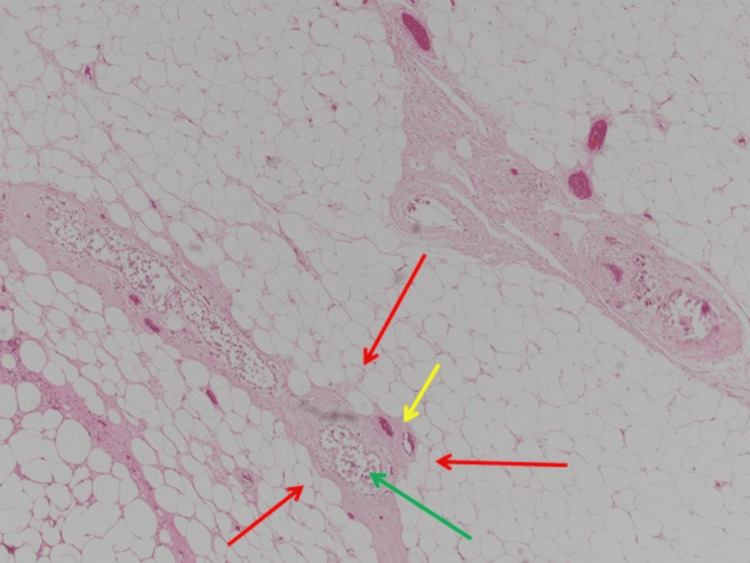
Breast tissue with calcifications in blood vessels (yellow arrow) and fat necrosis (red arrows) around breast ducts (green arrow) at x4 magnification.

Multiple skin punch biopsies showed features of acute and chronic inflammation with infection. The clinical, radiological, and histological findings were suggestive of calciphylaxis. Initially, it was managed with painkillers, antibiotics, and sodium thiosulfate. Then, bilateral simple mastectomies were performed as breast preservation was not an option due to a large necrotic area on the left side and potential disease progress on the contralateral side. The postoperative histology showed ulcerated breast skin with blood vessel wall calcifications and fat necrosis around breast ducts (Figures 5-7).

The dry ulceration on the right heel and right first toe was treated conservatively with regular clean dry wound dressing.

## Discussion

Calciphylaxis is also known as calcific uremic arteriolopathy, metastatic calcinosis cutis, and necrotizing or calcifying panniculitis. Its pathogenesis is unclear. Calcium deposits are seen in the medial layer of small blood vessels, such as arterioles and capillaries, in addition to intimal fibrosis. The progressive calcification and endothelial dysfunction result in thrombotic occlusion and subsequent ischemic necrosis of the soft tissue and skin [2,4]. The phenomenon itself was first described in 1898 by Bryant and White; however, it was not until 1962 that the term “calciphylaxis” was introduced by Hans Selye [1,4]. The pathogenesis of calciphylaxis is not well understood yet.

According to Selye's experimental animal models, the mechanism of disease development is theorized to be a three-stage hypersensitivity reaction: sensitization, the so-called critical period, and cutaneous calcinosis. The sensitization phase typically takes place with high parathyroid hormone levels, specifically in patients with end-stage kidney disease. The sensitizer could be vitamin D or the parathyroid hormone itself. The elapsed time between sensitization and calcinosis or challenge is called the “critical period.” The challenging agent, such as trauma, thrombosis, or hypotension, triggers the calcinosis phase. This process results in ischemic infarction, dystrophic calcification, and ulceration. It has been reported that, regardless of the cause, calciphylaxis is almost always associated with high levels of calcium-phosphate minerals [5,6].

Several authors have mentioned the abnormal biochemical environment seen in patients with this disease because hyperphosphatemia, hypercalcemia, and hyperglycemia promote the transformation of the smooth muscle cells of small blood vessels into osteoblast-like cells, resulting in vascular calcification [7]. In addition to the skin and subcutaneous tissue of the limbs, trunk, and buttocks, calciphylaxis as a systemic disease also affects the deeper organs, including the kidneys, lungs, brain, heart, and gastrointestinal tract. It is less frequently encountered in the penis, eyes, and breasts [2,8,9]. Calciphylaxis is usually associated with end-stage kidney disease, where patients are on long-term renal dialysis with subsequent secondary hyperparathyroidism. However, it is crucial to note that the presence of renal failure is not an absolute requirement in patients with calciphylaxis; this condition has been detected in patients without renal failure as well, which is also known as non-uremic calciphylaxis (NUC). The most frequently encountered conditions linked to NUC are hyperparathyroidism, alcoholic liver disease, malignant neoplasms, and autoimmune disorders (Table 1).

**Table 1:** Risk factors associated with calciphylaxis. DM: diabetes mellitus.

End‐stage kidney disease
Female gender
Caucasian race
Warfarin
Vitamin D treatment
Corticosteroids
Hypercalcemia
Hyperparathyroidism
Thiazide
Intravenous iron
Raised serum phosphate
Raised serum aluminum
Autoimmune disease
Obesity
Type II DM
Hypertension
Protein malnutrition
Hypercoagulable states
Humoral hypercalcemia of malignancy
Hereditary thrombophilia
Alcoholic liver disease
Protein C deficiency
Lupus anticoagulant

Certain medications may increase the risk of NUC, particularly warfarin. Matrix GLA protein (MGP) normally inhibits calcification of the arteries, and warfarin blocks vitamin K-dependent carboxylation of the MGP. This mechanism results in a reduction in the inhibition level of local calcification. The deficiency of other vascular calcification inhibitors, such as fetuin-A, may lead to the development of calciphylaxis. Fetuin-A is a glycoprotein; its role is to bind calcium and phosphorus and block the calcification of soft tissue and blood vessels [5,10]. Calciphylaxis triggers in the present case report were end-stage renal disease, female gender, high BMI, dialysis dependency, and hypertension. In patients with end-stage renal failure who are undergoing dialysis, the incidence of calciphylaxis ranges from 0.04% to 4% [7].

Cutaneous calciphylaxis is the most frequent form and has a tendency to occur at high-trauma sites such as the thighs, abdomen, buttocks, and breasts [6]. One of the common cutaneous physical signs is the presence of a painful, mottled, net-like cyanotic discolored skin rash that resembles livedo reticularis lesions. They may have a transient or persistent course; later, the lesions may progress to plaques, nodules, or bullae. By the time they grow and become more stellate, they progress into necrotic foci, eventually developing painful ulcerations [5,6]. Diagnosis can be challenging, and this condition can clinically resemble other dermatological diseases, in particular in non-uremic patients, which may include warfarin-associated skin necrosis and cellulitis (Table 2).

**Table 2:** Differential diagnoses of breast calciphylaxis.

Inflammatory breast cancer
Tuberculous mastitis
Vasculitis
Warfarin-associated skin necrosis
Antiphospholipid syndrome
Cholesterol embolization
Cellulitis
Ecthyma gangrenosum

An important differential diagnosis of this condition is breast malignancy, which is the most frequently diagnosed cancer globally. GLOBOCAN data published in 2020 have revealed that breast cancer is responsible for 12% of newly diagnosed cancer cases, followed by lung, colorectal, prostate, and gastric cancers [11-14]. At present, tissue diagnosis via skin biopsy is the most definitive method for diagnosing this condition. Microscopic examination can reveal intramural calcification and intimal fibrosis of small and medium blood vessels with the absence of vasculitis changes. Additionally, panniculitis, stromal dystrophic calcifications, and fat necrosis indicate the possibility of calciphylaxis. A characteristic feature of calciphylaxis is the presence of diffuse calcification of the capillaries in the adipose tissue, with calcified deposits composed of calcium and phosphorus [5,6,15-17].

Some research has reported that a deep punch biopsy may precipitate ulceration, pain, bleeding, necrosis, and sepsis; however, in one study, breast cancer was reported in a breast with calciphylaxis [18]. An appropriate imaging modality could also be helpful depending on the affected site. In our case, the breast mammogram revealed extensive vascular wall calcification; in some cases, widespread microcalcification could also be seen. Breast ultrasonography may reveal hypoechoic lesions without internal vascularity. In cases where calciphylaxis is suspected and the biopsy is not diagnostic or the site is inaccessible for biopsy, several authors have recommended the utilization of a suitable imaging modality.

For the diagnosis of calciphylaxis, whole-body bone scans can detect foci of subtle calcification features in cases where the affected organ is not accessible for biopsy and where technetium-99m methyl diphosphonate radiotracer uptake can be seen in subcutaneous tissues and other affected areas. In addition, computed tomography of the abdomen may reveal extensive intra-abdominal vascular calcification and subcutaneous calcium deposition in patients with suspected calciphylaxis. Plain X-rays and ultrasonography have also been used to detect subcutaneous vascular calcifications [19].

The management of calciphylaxis requires a multidisciplinary approach involving nephrology, wound care, dermatology, microbiology, surgery, and pain management specialists. Intravenous sodium thiosulfate, a potent antioxidant and chelating agent that increases the solubility of calcium minerals, has been used successfully in the management of calciphylaxis. It binds to calcium to form calcium thiosulfate, which is a highly soluble calcium salt [20]. In addition, the antioxidant properties of sodium thiosulfate help to reverse the endothelial dysfunction seen in calciphylaxis [6]. Bisphosphonates also have a place in calciphylaxis treatment. They act by modulating the effects of alkaline phosphatase on the vascular smooth muscle of small blood vessels, blocking the transport of phosphorus and thus resulting in a lower rate of calcium phosphate crystal formation [15]. These patients may also require effective pain management and risk factor control, such as discontinuing the use of warfarin and corticosteroids, in addition to correcting electrolyte disturbances and intensifying dialysis [15]. Furthermore, hyperbaric oxygen therapy is recommended as one of the temporary treatments for calciphylaxis to improve tissue oxygenation [5].

Surgical intervention is reserved for cases with acute wound infections, as they may need debridement, as well as for patients with painful progressive necrotic lesions in the breast, where a bilateral mastectomy can be performed for local pain control and to allow a better quality of life for the patient. For cases of calciphylaxis associated with secondary hyperparathyroidism, some researchers have suggested parathyroidectomy to keep calcium at lower levels [15]. The one-year survival rate for all cases with calciphylaxis has previously been estimated to be between 45.8% and 50%; this rate increases to 80% in patients with ulcerations. The poor prognosis linked to this condition is mainly due to the high risk of complications, especially sepsis [7,10]. 

## Conclusions

Breast calciphylaxis is a rare condition that must be considered a potential cause of atypical bilateral breast or nipple-areola complex ulceration, especially in patients with end-stage renal failure. This condition can mimic locally advanced breast cancer with extensive skin invasion; the appropriate imaging modality as well as a tissue biopsy can help in the diagnosis. A multidisciplinary approach is required in calciphylaxis management to facilitate a better quality of life for patients, and further research is needed to establish potential preventive measures in high-risk groups.
